# The origin of volatile elements in the Earth–Moon system

**DOI:** 10.1073/pnas.2115726119

**Published:** 2022-02-14

**Authors:** Lars E. Borg, Gregory A. Brennecka, Thomas S. Kruijer

**Affiliations:** ^a^Nuclear and Chemical Science Division, Lawrence Livermore National Laboratory, Livermore, CA 94550;; ^b^Department of Solar System, Impacts & Meteorites, Museum fur Naturkunde, Berlin 10115, Germany

**Keywords:** Moon, volatile elements, Giant Impact, Moon-forming impact

## Abstract

The origin of volatile species such as water in the Earth–Moon system is a subject of intense debate but is obfuscated by the potential for volatile loss during the Giant Impact that resulted in the formation of these bodies. One way to address these topics and place constraints on the temporal evolution of volatile components in planetary bodies is by using the observed decay of ^87^Rb to ^87^Sr because Rb is a moderately volatile element, whereas Sr is much more refractory. Here, we show that lunar highland rocks that crystallized ∼4.35 billion years ago exhibit very limited ingrowth of ^87^Sr, indicating that prior to the Moon-forming impact, the impactor commonly referred to as “Theia” and the proto-Earth both must have already been strongly depleted in volatile elements relative to primitive meteorites. These results imply that 1) the volatile element depletion of the Moon did not arise from the Giant Impact, 2) volatile element distributions on the Moon and Earth were principally inherited from their precursors, 3) both Theia and the proto-Earth probably formed in the inner solar system, and 4) the Giant Impact occurred relatively late in solar system history.

## Significance

Understanding the history of volatile species such as water in the Earth–Moon system is a major objective of planetary science. In this work, we use the moderately volatile element Rb, which has a long-lived isotope (^87^Rb) that decays to ^87^Sr, to show that lunar volatile element depletion was not caused by the Moon-forming impact. The Rb–Sr systematics of lunar rocks mandate that the bodies involved in the impact that formed the Earth–Moon system were depleted in volatile elements relative to the bulk solar system prior to the impact. As such, Earth’s relatively small proportion of water is either primarily indigenous or was added after the Giant Impact from a source that contained essentially no moderately volatile elements.

Understanding the formation of the Moon has long been a topic of intense interest, although hard constraints on this event only developed after the Apollo program returned lunar samples to Earth. Based on the thousands of lunar rocks that have been studied to date, arguably one of the most stringent of these constraints is that the Moon is strongly depleted in volatile elements relative to the solar photosphere, primitive meteorites, and Earth. Recognition of such a depletion of volatile elements, combined with the orbital mechanics of the Moon and geochemical evidence that it differentiated from a mostly molten state, led to the now widely accepted “Giant Impact” hypothesis, in which the Moon accreted from a volatile element–depleted debris disk produced by an impact between a Mars-sized body (Theia) and the proto-Earth (1). Yet, the formation of the Moon through such an impact scenario raises questions about the composition of the proto-Earth and Theia and their respective contributions to the makeup and subsequent evolution of the Earth–Moon system. Of particular interest is how and when the Moon and Earth obtained their present allotments of volatile components, including, and most importantly, water. Did the Moon and Earth form with their current allotments of volatile elements, or were these elements lost during the Giant Impact and reintroduced to Earth by later accretion of volatile element–laden materials? Here, we address this issue using the Rb–Sr isotopic systematics of lunar samples to provide time constraints on the history and distribution of volatile elements in the Earth–Moon system.

## Constraining Volatile Element Distribution through Rb–Sr Isotopic Evolution

**Fig. 1.**
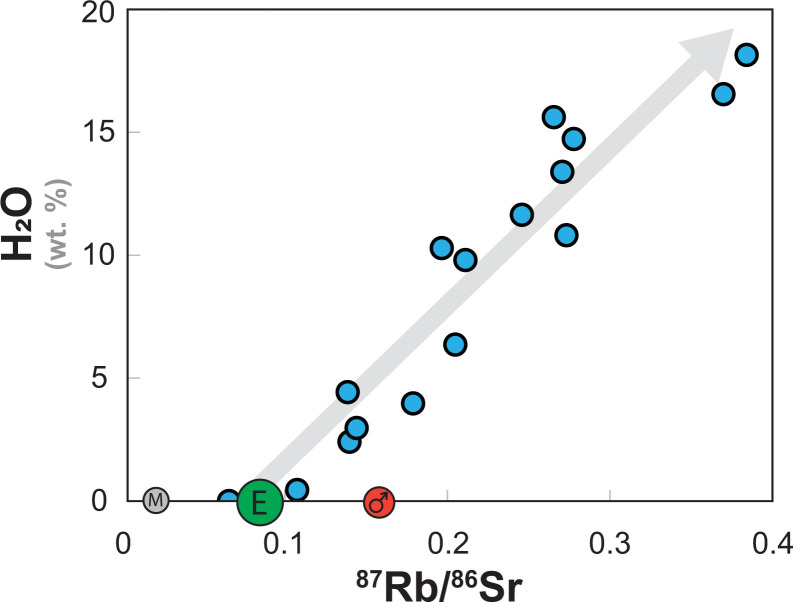
Plot of ^87^Rb/^86^Sr versus H_2_O contents of unprocessed primitive meteorites that serve as building blocks to terrestrial planetary bodies. The dark circle “E” represents Earth, the gray “M” represents the Moon and the red symbol represents Mars. The correlation demonstrates that ^87^Rb/^86^Sr is a good proxy for the abundance of highly volatile elements and volatile species. Data from Braukmüller et al. (3).

Since the late 1960s, research in lunar chronology has widely employed the Rb–Sr isotopic system, which is based on the decay of ^87^Rb to ^87^Sr (half-life = 49.4 Ga). The Rb–Sr system not only quantifies the time at which minerals in an igneous sample reached isotopic equilibrium (i.e., crystallized with the same ^87^Sr/^86^Sr ratio) but it also identifies the initial ^87^Sr/^86^Sr ratio of the parental magma at the time of crystallization. In turn, the initial ^87^Sr/^86^Sr of the parental magma can be used to determine the ^87^Rb/^86^Sr ratio of the sources from which the parental magmas were derived. This is a particularly powerful tool for understanding the volatile element history of a planetary body because Rb is a moderately volatile element with a 50% condensation temperature (T_50%_) of 800 K and is much less refractory than Sr (T_50%_ = 1455 K) (2). The ^87^Rb/^86^Sr ratio of an igneous system, therefore, acts as a general index for the abundances of volatile elements relative to refractory elements in that system. Thus, ^87^Rb/^86^Sr is an index of the temperature conditions during condensation of the materials that formed that body. Furthermore, the observed relationship between the measured ^87^Rb/^86^Sr and water content measured in primitive meteorites that serve as the building blocks of planetary bodies demonstrates that ^87^Rb/^86^Sr can also serve as a proxy for highly volatile element species in these bodies (3, Fig. 1). Finally, ^87^Rb continually decays to ^87^Sr so that the ^87^Sr/^86^Sr ratio of any material increases over time, cumulatively recording its volatile element history.

## Lunar Samples

To constrain the evolution of volatile elements in the Earth–Moon system using the Rb–Sr isotopic system, lunar rocks that represent the ^87^Sr/^86^Sr of the bulk Moon at a known time must be identified. Relatively young basaltic magmas are not ideal samples due to the fact that they experienced 0.5 to 1.5 Ga of Rb–Sr isotopic evolution in the interval between solidification of their lunar magma ocean (LMO) source regions and basalt crystallization on the lunar surface. Instead, ancient highland rocks, which formed at or very near the time of LMO solidification, are preferred because they did not experience significant Rb–Sr isotopic evolution after the Moon solidified. These rocks therefore preserve a record of the ^87^Sr/^86^Sr ratio of the bulk Moon at the time they crystallized, essentially providing a fiducial marker for the Rb content of lunar precursors. However, not all ancient highland samples are well suited for this purpose, as many of these samples have experienced post-crystallization metamorphism associated with impacts on the lunar surface, where Rb is mobilized and removed (4, 5). Consequently, the most reliable highland samples for this work are those that yield undisturbed Rb–Sr ages identified as concordant with ages determined by other chronometers such as ^147^Sm–^143^Nd in the same rocks.

One set of samples that meet these criteria are Mg-suite samples 67667, 76535, and 78238. Not only do these samples yield concordant Rb–Sr and Sm–Nd ages, their ancient crystallization ages of ∼4.30 to ∼4.35 Ga (Table 1) represent some of the oldest samples from the Moon, similar to ages determined for the formation of LMO cumulates that range from 4.34 to 4.38 Ga (*SI Appendix*). In addition, the initial Nd isotopic compositions (i.e., ε^143^Nd, see *Methods Summary*) of these samples suggest that they formed from minimally differentiated sources that were produced near the age of the primordial solidification of the Moon. Two additional rocks that have been dated using the Sm–Nd isotopic system are the ferroan anorthosite suite (FAS) samples 60016 and 60025. Like the Mg-suite samples discussed above, they have initial ^143^Nd/^144^Nd ratios indicating that they derive from undifferentiated or minimally differentiated sources. Furthermore, these FAS samples are thought to be primary crystallization products of the LMO. However, unlike the well-behaved Mg-suite samples, Rb–Sr ages of FAS rocks are disturbed due to the mobilization of Rb caused by impact metamorphism so that the initial ^87^Sr/^86^Sr of FAS samples can only be indirectly inferred using the Rb–Sr isotopic systematics of plagioclase mineral fractions and their determined Sm–Nd ages. Whereas the estimates of initial ^87^Sr/^86^Sr for FAS samples are very similar to those from Rb–Sr isochrons of Mg-suite rocks, it is important to consider that these estimates represent maximum values because metamorphism removes Rb from the plagioclase mineral fractions (4, 5). The isotopic characteristics of Mg-suite and FAS samples described above are taken from the literature and can be used to estimate the bulk ^87^Sr/^86^Sr of the Moon. The ages of these samples range from 4.302 ± 0.028 to 4.359 ± 0.003 Ga, and the initial ^87^Sr/^86^Sr ratios range from 0.69905 ± 0.00001 to 0.69912 ± 0.00001 (all data and references given in Table 1). Whereas all of these values comprehensively show that the Moon was significantly depleted in Rb relative to Sr, the lowest initial ^87^Sr/^86^Sr value from the FAS sample 60025 defines the maximum allowable ^87^Sr ingrowth and therefore a corresponding maximum Rb/Sr ratio for the Moon (Table 1). The observation that Mg-suite samples have ages and initial Sr isotopic compositions that are nearly identical to the FAS samples supports adopting 60025 to represent the bulk Moon Sr isotopic composition at 4,359 Ma.

**Table 1.** Initial ^87^Sr/^86^Sr and ages of highlands samples

Sample	Suite	Age (Ga)	Initial ^87^Sr/^86^Sr	Initial ɛ^143^Nd
67667	Mg-suite	4.352 ± 0.028	0.699116 ± 0.000010	0.04 ± 0.11
76535	Mg-suite	4.306 ± 0.010	0.699105 ± 0.000022	−0.15 ± 0.22
78236	Mg-suite	4.349 ± 0.019	0.699116 ± 0.000022	−0.27 ± 0.74
60016	FAS	4.302 ± 0.028	0.699062 ± 0.000011	−0.28 ± 0.14
60025	FAS	4.359 ± 0.003	0.699050 ± 0.000010	−0.29 ± 0.09

Data from refs. 40 to 45.

## Modeling Rb–Sr Isotopic Evolution

**Fig. 2.**
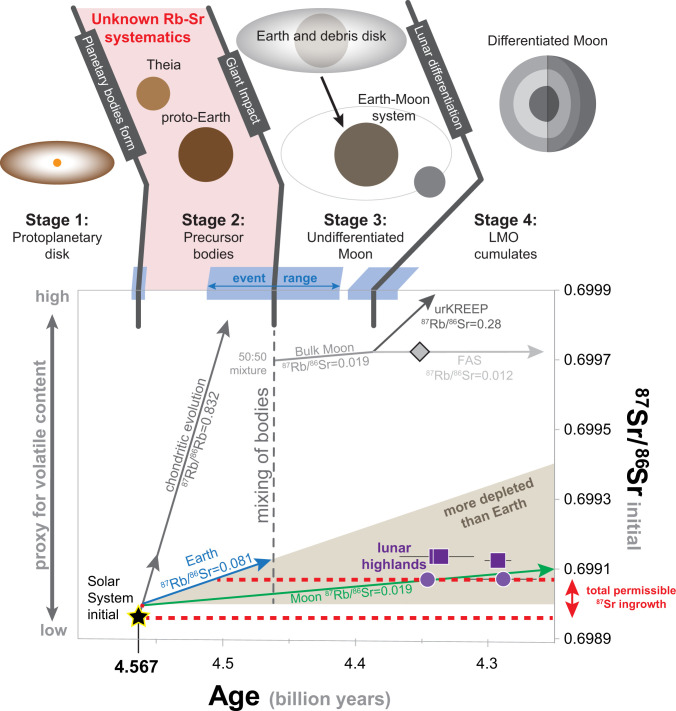
The four stages of Rb–Sr isotopic evolution of the Earth–Moon system. The timing of these stages is defined by ranges of ages from the literature, which are illustrated in blue at the top with the middle of each range selected for display purposes. FAS samples are purple circles, and Mg-suite samples are purple squares. Rb–Sr evolution is discussed in the main text. The total permissible radiogenic growth of ^87^Sr/^86^Sr from 4.56 to 4.36 Ga is 0.00007 (0.01%) and is represented by two horizontal dashed red lines. The gray diamond represents a modeled composition of the bulk Moon assuming Theia and proto-Earth had an ^87^Rb/^86^Sr of chondritic meteorites and present-day Earth, respectively, and were evenly mixed by the Giant Impact that occurred at 4.46 Ga (see text). Dark gray, blue, and green lines are ^87^Sr growth curves starting at 4.57 Ga assuming reservoirs with ^87^Rb/^86^Sr ratios of chondritic meteorites, bulk Earth, and bulk Moon, respectively.

Combining the lunar sample data with Rb–Sr isotopic evolution places constraints on the volatile element budget of the precursor materials involved in the formation of the Earth–Moon system (i.e., proto-Earth and Theia) and thus on the origin and timing of volatile elements on these bodies. This exercise requires knowing the formation ages of each reservoir involved, the duration each reservoir existed, and their respective ^87^Rb/^86^Sr ratios. Specifically, lunar Rb–Sr evolution can be considered to have occurred in four stages: 1) protoplanetary disk stage, 2) precursor bodies stage, 3) undifferentiated Moon stage, and 4) lunar magma ocean cumulates stage (Fig. 2). The first stage occurred in the protoplanetary disk before the formation of planetary bodies. This reservoir inherited the initial solar system ^87^Sr/^86^Sr value of 0.69898 at 4.567 Ga and evolved for 1 to 2 Ma (6, 7), with a solar (or chondritic) ^87^Rb/^86^Sr ratio represented by primitive CI-type chondritic meteorites (*SI Appendix*). The second stage of growth occurred in proto-Earth and Theia after they accreted from the protoplanetary disk as planetary bodies. These reservoirs existed until the Giant Impact, when proto-Earth and Theia were mixed and combined to form the Moon and Earth. The timing of the Giant Impact is estimated to range from 4.42 to 4.52 Ga (8–13); however, the exact age of the impact as well as the ^87^Rb/^86^Sr ratios of Theia and the proto-Earth are unknown and hence objectives of this investigation. The third stage of Rb–Sr isotopic evolution occurred after the Giant Impact in the lunar accretion disk and in the accreted, but yet undifferentiated, Moon. Previous studies have determined the average ^87^Rb/^86^Sr of the bulk Moon to be 0.019 ± 0.006, and this value is used in our calculations (*SI Appendix*). The final stage of evolution occurred within the cumulate rocks of the LMO. This final stage of Rb–Sr evolution is not modeled independently from Stage 3 because it contributed negligibly to the overall Rb–Sr systematics of the highland samples. Ferroan anorthosite suite samples are LMO cumulates, so their initial Sr isotopic compositions directly record the composition of the LMO and, hence, the bulk Moon, at the time they formed. Furthermore, the ^87^Rb/^86^Sr of FAS samples are not significantly different from the ^87^Rb/^86^Sr estimated from the bulk Moon, and thus, Sr growth is expected to be similar in both reservoirs.

The initial ^87^Sr/^86^Sr of the bulk Moon depends on three parameters: 1) the ^87^Rb/^86^Sr of both Theia and the proto-Earth, 2) the proportions of Theia and the proto-Earth comprising the Moon, and 3) the timing of the Giant Impact as illustrated in Fig. 2. When exemplary calculations using endmember compositions of these estimated parameters are made, important conclusions can be drawn, even when the parameters are poorly known. The details of the calculations are presented in *SI Appendix*, Table S1. For example, if Theia and the proto-Earth had ^87^Rb/^86^Sr values similar to chondritic meteorites (0.832 ± 0.028) and the present-day Earth (0.081 ± 0.009, *SI Appendix*), respectively, and were mixed in equal proportions in the Moon following the Giant Impact modeled at 4.46 Ga (gray lines on Fig. 2), then the bulk Moon would have an ^87^Sr/^86^Sr of 0.69975 at 4.36 Ga (gray diamond in Fig. 2 and *SI Appendix*, Table S1, Model 1). This value is significantly higher than the initial values observed in the ∼4.36-Ga highland samples (0.69905 to 0.69912; Table 1), making this scenario unfeasible. On the other hand, if both Theia and the proto-Earth evolved with ^87^Rb/^86^Sr similar to the present-day bulk Moon (0.019 ± 0.006, which is significantly more depleted than present-day bulk Earth), the Moon would have an ^87^Sr/^86^Sr of 0.69906 at 4.36 Ga, which is indistinguishable from the lowest highland sample 60025 (green line in Fig. 2 and *SI Appendix*, Table S1, Model 2).

**Fig. 3.**
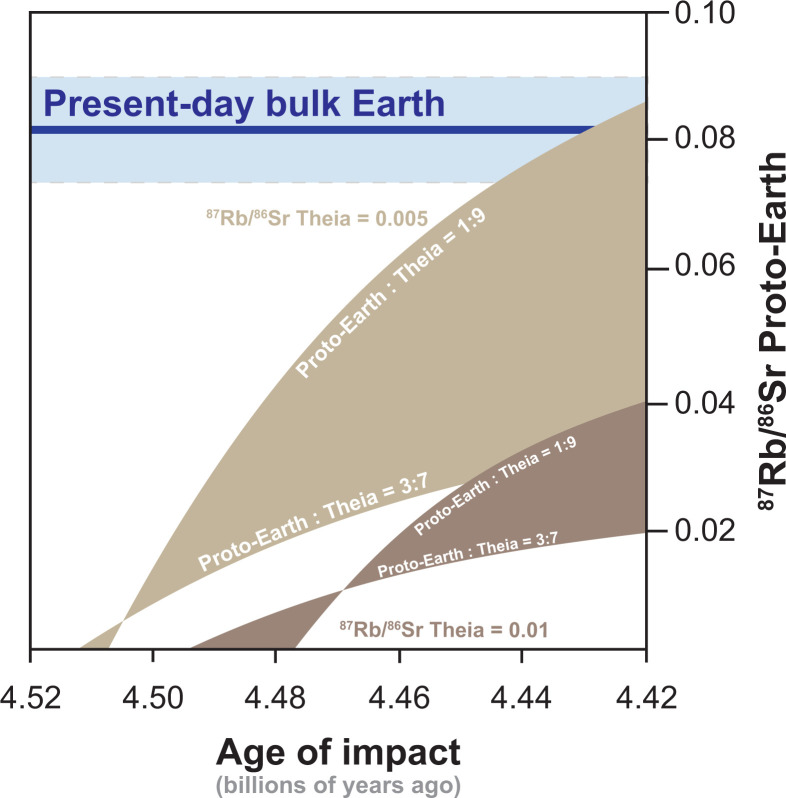
Plot of ^87^Rb/^86^Sr of the proto-Earth versus the age of the Giant Impact. The isotopic composition of the proto-Earth is calculated using the approach outlined in the text and in *SI Appendix*. Theia is assumed to have an ^87^Rb/^86^Sr of 0.005 (light brown field) and 0.010 (dark brown field). The upper bound of each field illustrates the case in which the Moon comprises 90% Theia and 10% proto-Earth. The lower bound depicts the case in which the Moon comprises 70% Theia and 30% proto-Earth, and it was chosen because it illustrates that a relatively modest decrease in the proportion of Theia in the Moon dramatically lowers the ^87^Rb/^86^Sr calculated for the proto-Earth. Crossover points represent cases in which the ^87^Rb/^86^Sr of proto-Earth is equal to that of Theia.

These examples demonstrate that the Moon and proto-Earth must be derived from precursors that were already very strongly depleted in volatile elements shortly after the beginning of the solar system. Furthermore, scenarios in which Theia and the proto-Earth had elevated ^87^Rb/^86^Sr ratios that were subsequently lowered during volatile element loss after the Giant Impact are not permissible because even a few million years of ^87^Rb decay in volatile element–enriched reservoirs would have produced enough ^87^Sr to exceed the observed initial ^87^Sr/^86^Sr ratios of the highland samples (Fig. 2 and *SI Appendix*, Table S1, Model 3). This contradicts Rb–Sr isotopic modeling of FAS samples completed by ref. 14 because they used whole-rock Rb–Sr isotopic measurements and mistakenly assumed a crystallization age of these samples of 4.52 Ga (*SI Appendix*). However, this observation does not require both Theia and the proto-Earth to be equally depleted in volatile elements; it is possible that one precursor body could have been slightly less depleted in volatile elements provided the other body was strongly depleted in volatile elements and contributed the bulk of the material that formed the Moon. Thus, if the Moon simply inherited its low ^87^Rb/^86^Sr from Theia, the proto-Earth might have a proportionally higher ^87^Rb/^86^Sr corresponding to more elevated volatile element abundances in Earth relative to the Moon. With the knowledge of the initial ^87^Sr/^86^Sr of the lunar highland samples derived here, it is possible to constrain the allowable ^87^Rb/^86^Sr of the proto-Earth and Theia by assuming variable proportions of Theia and the proto-Earth comprising the Moon and the timing of the Giant Impact (Fig. 3).

One endmember scenario that maximizes the ^87^Rb/^86^Sr ratio of the proto-Earth assumes Theia had no volatile elements (^87^Rb/^86^Sr = 0) and that the Moon contained the largest (90%) proportion of Theia (15, 16). In this case, the ^87^Rb/^86^Sr of the proto-Earth is calculated to range from 0 (*SI Appendix*, Table S1, Model 4) to 0.13 (*SI Appendix*, Table S1, Model 5) depending on whether the Giant Impact occurred at 4.52 or 4.42 Ga, respectively. Even in this unrealistic scenario in which Theia had zero Rb, the calculated ^87^Rb/^86^Sr value of proto-Earth is well below estimates for chondritic meteorites (0.832), confirming that Theia and the proto-Earth were both strongly depleted in volatile elements. A more reasonable approach diagramed in Fig. 3 assumes Theia had an ^87^Rb/^86^Sr of 0.005, approximately the value of the most volatile element–depleted igneous samples known in the solar system, angrite meteorites. This output shows that, even assuming Theia was equivalent to the most volatile-depleted body known, either the proto-Earth must have had a lower ^87^Rb/^86^Sr than the Earth does today or that the Giant Impact occurred no earlier than 4.43 Ga (Fig. 3 and *SI Appendix*, Table S1, Model 6). Note that if the Moon is comprised of a greater proportion proto-Earth, say only 70% Theia (17–20), then the proto-Earth must have an even lower ^87^Rb/^86^Sr of ≤0.04 (*SI Appendix*, Table S1, Model 7). For a more volatile element–rich scenario for Theia, we assume an ^87^Rb/^86^Sr of 0.01, roughly half that observed in the bulk Moon (^87^Rb/^86^Sr = 0.019). In this case, the proto-Earth must also be strongly depleted in volatile elements and have an ^87^Rb/^86^Sr that is ≤0.04 (Fig. 3 and *SI Appendix*, Table S1, Model 8).

## Ramification of Modeling

All Giant Impact mixing scenarios outlined in the calculations presented in *SI Appendix* and shown in Fig. 3 require exceedingly low ^87^Rb/^86^Sr for both Theia and the proto-Earth, and this places several important constraints on hypotheses for the origin and evolution of the Moon and Earth. Since most model solutions require the proto-Earth to have an ^87^Rb/^86^Sr that was substantially less than present-day Earth, the first important implication of volatile element–depleted precursors relates to the ultimate source of extant volatile elements on Earth. For example, if Theia had an ^87^Rb/^86^Sr of 0.005 and made up 90% of the Moon and the Giant Impact occurred at 4.49 Ga (*SI Appendix*, Table S1, Model 9), then the proto-Earth would have an ^87^Rb/^86^Sr of ∼0.025 (Fig. 3), which corresponds to only ∼17% of its current allotment of Rb. Thus, the addition of volatile-rich material after the Giant Impact must account for the majority, in this case 83%, of the moderately volatile elements present in modern Earth. However, the addition of such a large amount of chondritic material after the Giant Impact is inconsistent with the abundances of highly siderophile elements in terrestrial rocks. In fact, mass balance calculations suggest that the addition of this late veneer after the Giant Impact cannot account for more than ∼0.5% of Earth’s mass (21–24) and would contribute negligibly to Earth’s Rb budget. This conundrum can be reconciled by model solutions that adopt Earth-like ^87^Rb/^86^Sr for the proto-Earth, requiring no significant addition of Rb to Earth after the Giant Impact. In this scenario, Theia must have had a very low ^87^Rb/^86^Sr (∼0.005) and made up a large proportion of the Moon (∼90%), and the Giant Impact occurred relatively late (after 4.43 Ga) in solar system history (Fig. 3 and *SI Appendix*, Table S1, Model 6).

The described scenario in which both the proto-Earth and Theia were volatile element depleted (low ^87^Rb/^86^Sr) has major implications for the formation location of these bodies. Protoplanetary disks have strong thermal gradients (25, 26), and this thermal gradient is thought to be responsible for the compositional gradient by which volatile element abundances increase the further away a material forms from the Sun (e.g., refs. 27 and 28). Thus, the fact that both Theia and the proto-Earth have low ^87^Rb/^86^Sr suggests they formed near one another in the inner solar system. In addition to an elemental gradient, the isotopic compositions of elements such as Cr, Ti, Zr, Mo, and Nd also demonstrate spatial variation in the solar system, reflecting variable distributions of isotopically anomalous components with heliocentric distance (e.g., refs. 29 to 34). Therefore, the formation of both Theia and the proto-Earth in the inner solar system also accounts for the fact that Earth and Moon have nearly identical O, Ti, and Cr isotopic compositions, obviating the need to explain these isotopic similarities with the re-equilibrium of Earth and Moon after the Giant Impact (35, 36) or the mixing of Theia and the proto-Earth in near equal proportions following the Giant Impact (17–20). Instead, Theia and the proto-Earth were composed of isotopically similar materials, as suggested by refs. 37 and 38, because they both originated in the inner solar system.

The observed correlation between ^87^Rb/^86^Sr and water content in primitive meteorite building blocks of terrestrial bodies suggest the relative abundances of moderately volatile elements such as Rb inform the behavior of more volatile species like water (*SI Appendix*). Specifically, the low ^87^Rb/^86^Sr required for Theia and the proto-Earth suggest both of these bodies were depleted in water relative to primitive meteorites prior to the Giant Impact. The inability to account for low ^87^Rb/^86^Sr in the Earth–Moon system by volatile element loss associated with the Giant Impact combined with the fact that Theia and the proto-Earth have different ^87^Rb/^86^Sr ratios suggests that the higher water content of Earth relative to the Moon could be inherited from their precursor bodies. This is supported by modeling results that indicate that the proto-Earth could have had an ^87^Rb/^86^Sr ratio that was similar to that observed in the present-day Earth, implying the Earth could also have formed with an allotment of water that was not substantially different from what is observed today.

Although this work challenges the canonical presumption that volatile element depletion of the Moon occurred during the Giant Impact (39–41), it offers considerable advantages on the origin of volatiles in the Earth–Moon system. Based on the most likely scenario anchored by Rb–Sr systematics, the Giant Impact happened comparatively late (after 4.45 Ga), and the Moon formed largely (∼90%) from a strongly volatile-depleted Theia. This is consistent with the gradient of volatile elements observed in solar system materials, it accounts for the difference in the proportion of volatile elements in the Moon and Earth, and it explains the isotopic similarities in the Earth–Moon system. Furthermore, this model is consistent with calculations that suggest the Moon is composed mostly of Theia (15, 16) as well as some independent estimates for the timing of the Giant Impact after ∼4.43 Ga (9, 13). Lastly, the presented scenario suggests that the Earth and Moon formed with roughly the same budget of volatiles present today, and large amounts of such species are not required to be added to the Earth after the Giant Impact.

## Methods Summary

### Rb–Sr Isotopic Data.

The Rb–Sr isotopic data were culled from previous investigations (42–46) and presented in Table 1. The initial ^87^Sr/^86^Sr for Mg-suite rocks were calculated from Rb–Sr isochron regressions that yielded slopes corresponding to ages that were concordant with ages determined on the same mineral fractions using the Sm–Nd isotopic system. The ages presented in Table 1 are the weighted average of all concordant measured ages. The data for FAS are calculated from Rb–Sr data obtained from plagioclase mineral fractions using the Sm–Nd ages. All data are normalized to the NBS-987 Sr isotopic standard ^87^Sr/^86^Sr of 0.710250.

### Sm–Nd Isotopic Data.

The initial ɛ^143^Nd of the samples are derived from the *y*-intercept of regressions through mineral fraction data on Sm–Nd isochrons. The ɛ^143^Nd illustrates the deviation of the initial ^143^Nd/^144^Nd from an idealized undifferentiated reservoir with Sm–Nd isotopic systematics of chondritic meteorites measured by ref. 46.

The ɛ^143^Nd was calculated using the following equation:

$$\varepsilon^{143}\text{Nd}=\left[\frac{{\left(\frac{{}^{143}\text{Nd}}{{}^{144}\text{Nd}}\right)}_{\mathrm{Isochron}}^{TI}}{{\left(\frac{{}^{143}\text{Nd}}{{}^{144}\text{Nd}}\right)}_{\mathrm{Chondrite}}^{TI}}-1\right]\times 10^{4}.$$

The initial ɛ^143^Nd calculated from the Sm–Nd isochron of these highland rocks are within uncertainty, or very close to 0, indicating that these they were derived from precursor materials with ^147^Sm/^144^Nd that were similar to undifferentiated chondritic meteorites (47). The sources of these samples therefore must either be derived from sources that formed close to the crystallization age of the samples or have experienced only minimal amounts of melting and fractional crystallization. In addition, the ages determined for these samples are very close to ^146^Sm–^142^Nd ages determined for the solidification of the LMO of 4.336 ± 0.030 and model ages for late-stage crystallization products (urKREEP) of the magma ocean, which range from 4.35 to 4.38 Ga (*SI Appendix*).

## Supplementary Material

Supplementary File

## Data Availability

All study data are included in the article and/or *SI Appendix*.

## References

[1] W. Hartmann, D. Davis, Satellite-sized planetesimals and lunar origin. Icarus 24, 504–514 (1975).

[2] K. Lodders, Solar system abundances and condensation temperatures of the elements. Astrophysics J. 591, 1220–1247 (2003).

[3] N. Braukmüller, F. Wombacher, D. Hezel, R. Escoube, C. Münker, The chemical composition of carbonaceous chondrites: Implications for volatile element depletion, complementarity and alteration. Geochimica et Cosmochimica Acta. 239, 17–48 (2018).

[4] L. Borg , Isotopic studies of ferroan anorthosite 62236: A young lunar crustal rock from a light rare-earth element-depleted source. Geochimica et Cosmochimica Acta 63, 2679–2691 (1999).

[5] A. Gaffney, L. Borg, Y. Asmeron, C. Shearer, P. Burger, Disturbance of isotopic systematics during experimental shock and thermal metamorphism on a lunar basalt with implications for martian meteorite chronology. Meteoritics Planetary Sci. 46, 35–52 (2011).

[6] J. Chambers, Planetary accretion in the inner solar system. Earth Planetary Sci. Lett. 223, 241–252 (2004).

[7] U. Hans, T. Kleine, B. Bourdon. Rb–Sr chronology of volatile depletion in differentiated protoplanets: BABI, ADOR and ALL revisited. Earth Planetary Sci. Lett. 374, 204–214 (2013).

[8] T. Kruijer, C. Burkhardt, G. Budde, T. Kleine, Age of Jupiter inferred from the distinct genetics and formation times of meteorites. Proc. Natl. Acad. Sci*. U. S. A.* 114, 6712–6716 (2017). 28607079 10.1073/pnas.1704461114 PMC5495263

[9] J. Connelly, M. Bizzarro, Lead isotope evidence for a young formation age of the Earth-Moon system. Earth Planetary Sci. Lett. 452, 36–43 (2012).

[10] W. Bottke Dating the Moon-forming impact event with asteroidal meteorites. Science 348, 321–323 (2015). 25883354 10.1126/science.aaa0602

[11] T. Kruijer, T. Kleine, Tungsten isotopes and the origin of the Moon. Earth Planetary Sci. Lett. 475, 15–24 (2017).

[12] M. Thiemens, P. Sprung, R. Fonseca, F. Leitzke, C. Münker, Early Moon formation inferred from hafnium–tungsten systematics. Nat. Geosci. 12, 696–700 (2019). 10.1038/s41561-019-0398-3 PMC7617097 39649009

[13] M. Maurice, N. Tosi, S. Schwinger, D. Breuer, T. Kleine, A long-lived magma ocean on a young Moon. Sci. Adv. 6, eaba8949 (2020). 32695879 10.1126/sciadv.aba8949 PMC7351470

[14] A. Halliday, D. Porcelli, In search of lost planets–the paleocosmochemistry of the inner solar system. Earth Planetary Sci. Lett. 192, 545–559 (2001).

[15] R. Canup, Simulations of a late lunar-forming impact. Icarus 168, 433–456 (2004).

[16] R. Canup, Lunar-forming collisions with pre-impact rotation. Icarus 196, 518–538 (2008).

[17] R. Canup, Forming a Moon with an earth-like composition via a giant impact. Science 338, 1052–1055 (2012). 23076098 10.1126/science.1226073 PMC6476314

[18] M. Ćuk, S. Stewart, Making the Moon from a fast-spinning Earth: A giant impact followed by resonant despinning. Science 338, 1047–1052 (2012). 23076099 10.1126/science.1225542

[19] J. Zhang, N. Dauphas, A. Davis, I. Leya, A. Fedkin, The proto-Earth as a significant source of lunar material. Nat. Geosci. 5, 251–255 (2012).

[20] E. Young Oxygen isotopic evidence for vigorous mixing during the Moon-forming giant impact. Science 351, 493–496. (2016). 26823426 10.1126/science.aad0525

[21] R. Walker, Highly siderophile elements in the Earth, Moon and Mars: Update and implications for planetary accretion and differentiation. Chem. Erde Geochem*ica* 69, 101–125 (2009).

[22] U. Mann, D. Frost, D. Rubie, H. Becker, A. Audetat, Partitioning of Ru, Rh, Pd, Re, Ir and Pt between liquid metal and silicate at high pressures and high temperatures: Implications for the origin of highly siderophile element concentrations in the Earth’s mantle. Geochimica et Cosmochimica Acta 84, 593–613 (2012).

[23] Z. Wang, H. Becker, Ratios of S, Se and Te in the silicate Earth require a volatile-rich late veneer. Nature 499, 328–331 (2013). 23868263 10.1038/nature12285

[24] T. Kruijer, T. Kleine, M. Fischer-Gödde, P. Sprung, Lunar tungsten isotopic evidence for the late veneer. Nature 520, 534–537 (2015). 25855296 10.1038/nature14360

[25] A. Boss, Temperature is protoplanetary disks. Ann. Rev. Earth Planetary Sci. 26, 53–60 (1998).

[26] J. Chambers, An analytical model for an evolving protoplanetary disk with a disk wind. Astrophysical J. 879, 98 (2019).

[27] F. Ciesla, N. Cuzzi, The evolution of the water distribution in a viscous protoplanetary disk. Icarus 181, 178–204 (2006).

[28] J. Moriarty, N. Madhusudhan, D. Fischer, Chemistry in an evolving protoplanetary disk: Effects on terrestrial planet composition. Astrophysical J. 787, 81 (2014).

[29] A. Trinquier Origin of nucleosynthetic isotope heterogeneity in the solar protoplanetary disk. Science 324, 374–376 (2009). 19372428 10.1126/science.1168221

[30] L. Qin, C. Alexander, R. Carlson, M. Horan, T. Yokoyamo, Contributions to chromium isotopic variation of meteorites. Geochemica et Cosmochemica Acta 74, 1122–1145 (2010).

[31] T. Kruijer, T. Kleine, L. Borg, The great isotopic dichotomy of the early solar system. Nat. Astronomy 4, 32–40 (2020). 10.1038/s41550-019-0959-9 PMC7617084 39655089

[32] J. Render, G. Brennecka, Isotopic signatures as tools to reconstruct the primordial architecture of the solar system. Earth Planetary Sci. Lett. 555, 116705 (2021).

[33] P. Bonnand, J. Parkinson, M. Anand, Mass dependent fractionation of stable chromium isotopes in mare basalts: Implications for the formation and the differentiation of the Moon. Geochimica et Cosmochimica Acta. 175, 208–221 (2016).

[34] M. Millet , Titanium stable isotope investigation of magmatic processes on the Earth and Moon. Earth Planetary Sci. Lett. 449, 197–205 (2016).

[35] K. Pahlevan, D. Stevenson, Equilibration in the aftermath of the lunar-forming giant impact. Earth Planetary Sci. Lett. 262, 438–449 (2007).

[36] S. Lock The origin of the Moon within a terrestrial synestia. J. Geophys. Res. 123, 10 (2018).

[37] N. Dauphas, The isotopic nature of the Earth’s accreting material through time. Nature 541, 521–524 (2017). 28128239 10.1038/nature20830

[38] A. Mastrobuono-Battisti, H. B. Perets, S. N. Raymond. A primordial origin for the compositional similarity between the Earth and the Moon. Nature 520, 212 (2015). 25855458 10.1038/nature14333

[39] R. Canup, C. Visscher, J. Salmon, B. Fegley, Lunar volatile depletion due to incomplete accretion within an impact-generated disk. Nat. Geosci. 8, 918–921 (2015). 31360221 10.1038/ngeo2574 PMC6662721

[40] S. Charnoz, C. Michaut, Evolution of the protolunar disk: Dynamics, cooling timescale and implantation of volatiles onto the Earth. Icarus 260, 440–463 (2015).

[41] S. Charnoz , Tidal pull of the Earth strips the proto-Moon of its volatiles. Icarus 364, 114451 (2021).

[42] L. Borg, J. Connelly, M. Boyet, R. Carlson, Evidence that the Moon is either young or did not have a global magma ocean. Nature 477, 70‒72 (2011). 21849974 10.1038/nature10328

[43] L. Borg, J. Connelly, W. Cassata, A. Gaffney, M. Bizzarro, Chronologic implications for slow cooling of troctolite 76535 and temporal relationships between the Mg-suite and the ferroan anorthosite suite. Geochimica et Cosmochimica Acta 201, 377–391 (2017).

[44] L. Borg, W. Cassata, J. Wimpenny, A. Gaffney, C. Shearer, The formation and evolution of the Moon’s crust inferred from the Sm-Nd isotopic systematics of highlands rocks. Geochimica et Cosmochimica Acta 290, 312–320 (2020).

[45] J. Edmunson, L. Borg, L. Nyquist, Y. Asmeron, A combined Sm–Nd, Rb–Sr, and U–Pb isotopic study of Mg-suite norite 78238: Further evidence for early differentiation of the Moon. Geochimica et Cosmochimica Acta 73, 514–527 (2009).

[46] N. Marks, L. Borg, C. Shearer, W. Cassata, Geochronology of an Apollo 16 clast provides evidence for a basin‐forming impact 4.3 billion years ago. J. Geophys. Res. Planets 124, 2465–2481 (2019). 31894195 10.1029/2019JE005966 PMC6919926

[47] A. Bouvier, J. Vervoort, P. Patchett, The Lu–Hf and Sm–Nd isotopic composition of CHUR: Constraints from unequilibrated chondrites and implications for the bulk composition of terrestrial planets. Earth Planetary Sci. Lett. 273, 48–57 (2008).

