# Supplementary material for: The origin of volatile elements in the Earth–Moon system
Source: Proc Natl Acad Sci U S A. 2022 Feb 14;119(8):e2115726119. doi: 10.1073/pnas.2115726119 (PMC8872726; doi:10.1073/pnas.2115726119)
Supplement: Supplementary File [file pnas.2115726119.sapp.pdf]

PNAS
www.pnas.org

Supplementary Information for

The Origin of Volatile Elements in the Earth-Moon System

Lars E. Borg, Gregory A. Brennecka, Thomas S. Kruijer

Lars E, Borg
Email: borg5@llnl.gov

This PDF file includes:

Supplementary text
Figure S1
Table S1
SI References

Supplementary Information Text

Compilation of Ages of LMO cumulates

Lunar magma ocean (LMO) cumulates are divided into three main geochemical types. The first are mafic cumulates, produced in the early to mid-stage of LMO solidification (e.g., 1). These are the source regions of the mare basalts. The second group of LMO cumulates are intrusive crustal rocks of the ferroan anorthosite suite (FAS). These rocks crystallized from the LMO after about 80% solidification, floating to the surface to form much of the lunar crust. The third crystallization product of the LMO is urKREEP. This material represents the last vestiges of LMO solidification and is consequently characterized by strong enrichment in incompatible elements such as, K, REE, and P.

Formation ages for the mare basalt source regions, FAS, and urKREEP are the basis by which the solidification age of the Moon is estimated. Detailed reviews of these ages, their merits, and their inconsistencies are provided in (2-4). The formation age of the mare basalt source region has been measured by several groups using the ^{146}Sm-^{142}Nd isotopic system (5-9). The weighted average of these ages is 4333 ± 8 Ma (95% confidence, MSWD = 1.5). The uncertainty on the ^{146}Sm half-life is relatively large (10), and if included in the age calculation expands the uncertainty to 4333 ± 30 Ma.

Ages for various FAS samples have been determined using the Sm-Nd isotopic system by several groups. This system provides the most reliable ages because it is significantly more resistant to disturbance by impact metamorphism than other isotopic systems used to date planetary materials (3, 11). Nevertheless, the Sm-Nd age determinations are much less consistent than the ages determined for the mare basalt source regions ranging from 4576 ± 160 Ma (12) to 4302 ± 28 Ma (13). However, most recent FAS ages, including 60025 (4367 ± 11 Ma; 14), 60016 (4302 ± 28 Ma; 13), and 62237 (4350 ± 73 Ma; 15), cluster on the young side of this range. The weighted average of these FAS Sm-Nd ages, including Y86032 (4438 ± 34 Ma; 16) is 4365 ± 46 Ma (95% confidence, MSWD = 11.5).

Various types of isotope evolution models have been used to define the age of urKREEP formation. The Rb-Sr, Sm-Nd, and Lu-Hf isotopic systems have been applied to analyses of whole rock samples. The Rb-Sr system has yielded an age of 4.42 ± 0.07 Ga (17), Sm-Nd system has yielded ages of 4.36 ± 0.06 Ga (18) and 4.368 ± 0.026 Ga (19), and Lu-Hf system an age of 4.353 ± 0.037 Ga (19). This approach has also been applied to lunar zircons (20) but does not define a coherent age due to significant scatter of the data (21) and is consequently not included in the weighted average age of 4.367 ± 0.019 Ga (95% confidence, MSWD = 0.98).

Compilation of ^{87}Rb/^{86}Sr ratios of Solar System reservoirs

The elemental compositions of various Solar System reservoirs were compiled by (22) and used to calculate the ^{87}Rb/^{86}Sr of various Solar System reservoirs adopted by the Rb-Sr isotopic evolution models presented here. The bulk Moon is estimated to have ^{87}Rb/^{86}Sr of 0.016 (23), 0.027 (24), and 0.018 (25) yielding an average value of 0.019 ± 0.006. The bulk Earth is estimated to have ^{87}Rb/^{86}Sr of 0.070 (26), 0.087 (27), 0.089 (28), and 0.077 (29) yielding an average value of 0.081 ± 0.009. The average ^{87}Rb/^{86}Sr for primitive CI-type chondritic meteorites calculated from the values of 0.852 (30) and 0.812 (31) is 0.832 ± 0.028.

Modeling ^{87}Sr/^{86}Sr isotopic evolution of the Moon

The isotopic evolution of the Moon is considered to have occurred in four stages. These are discussed in the text and are illustrated in Figure 1. Briefly, the first stage (protoplanetary disk stage) begins with the formation of the first solids in the Solar System at 4567 Ma and ends with formation of Theia and the proto-Earth and dissipation of the protoplanetary disk at 4565 Ma. The second stage (the precursor bodies stage) involves isotopic evolution in Theia and proto-Earth from 4565 Ma and ends with the Giant Impact. The timing of the Giant Impact is unknown, so ages reported in the literature that range from 4520 to 4420 Ma are used in the calculations. The third stage of evolution (the undifferentiated Moon stage) occurs in the undifferentiated Moon starting

immediately after the Giant Impact until the Rb and Sr are fractionated during solidification of the Moon. Fractionation of Rb from Sr most likely occurs after about 70-80 percent solidification of the lunar magma ocean (LMO) when plagioclase begins to crystalize. Rubidium and strontium are incompatible in olivine and orthopyroxene, which are the initial crystallization products of the LMO, and so that crystallization of these phases will not alter the Rb/Sr of the evolving magma ocean significantly. The final stage of evolution occurs in the source regions of FAS and Mg-suite rocks. This stage is not modeled separately because FAS samples are thought to be crystallization products of the LMO so that their source is the bulk undifferentiated Moon. Evidence for this is derived from the observation that FAS sample 60025 has a crystallization age that matches the majority of ages determined for other LMO crystallization products including mare basalt source regions and urKREEP (see above). Furthermore, the ^{146}Sm-^{142}Nd systematics of 60025 are identical to the mare basalts indicating that the mare basalt sources and FAS sample 60025 evolved from the same chondritic reservoir at the same time as expected if both rock types represented primary magma ocean cumulates (32). Finally, it should be noted that the ^{87}Rb/^{86}Sr of the FAS suite of rocks is relatively close to the ^{87}Rb/^{86}Sr of the bulk Moon (33), so that the final stage of evolution in FAS samples would mimic evolution in the bulk Moon.

Ferroan anorthosite suite sample 60025 is the most accurately and precisely dated FAS sample because the age was obtained from three concordant chronometers including U-Pb (34). It has the lowest initial ^{86}Sr/^{86}Sr and is therefore used to define the end of the third stage of Sr isotopic evolution at 4359 Ma. The initial ^{87}Sr/^{86}Sr of 60025 is calculated from the Rb-Sr isotopic systematics of a plagioclase mineral fraction and the weighted average of the U-Pb and Sm-Nd age of the sample of 4359 Ma. The ^{87}Rb/^{86}Sr ratios of the reservoirs in which the ^{87}Sr/^{86}Sr evolves must also be estimated. The proto-planetary disk is estimated to have the ^{87}Rb/^{86}Sr represented by the average values measured in primitive chondritic meteorites of 0.832 (see above). The ^{87}Rb/^{86}Sr of the bulk Moon is taken from the average estimated value of 0.019 (see above). The ^{87}Rb/^{86}Sr of Theia and proto-Earth are the objectives of this modeling, so are varied in the calculations to fit the ^{87}Sr/^{86}Sr data from the initial ^{87}Sr/^{86}Sr of sample 60025.

The isotopic evolution of Sr is modeled progressively in the three stages outlined above using the equation:

$$\frac{^{87}Sr}{^{86}Sr} = I_{Sr} + \frac{^{87}Rb}{^{86}Sr}\left(e^{\lambda T_0} - e^{\lambda T_1}\right)$$

The ^{87}Sr/^{86}Sr calculated from this equation in the first stage is used as I_{Sr} in the next stage of the calculation. The initial starting value for ^{87}Sr/^{86}Sr is the Solar System initial value of 0.698980 value of (35). The ^{87}Rb/^{86}Sr used to calculate the ^{87}Sr/^{86}Sr at the end of stage 1 is 0.832, T_0 is 4567 Ma, T_1 is 4565 Ma, and λ is 1.402 x10^{-11} years. Because Theia and the proto-Earth formed separately in stage 2, they are modeled independently with variable ^{87}Rb/^{86}Sr ratios. Mixing of Sr from Theia and the proto-Earth during the Giant Impact assumes equal abundances of Sr in both bodies. After mixing Sr isotopic growth is assumed to occur in the bulk Moon with an ^{87}Rb/^{86}Sr of 0.019

Table S1 presents the results of several Sr isotope evolution scenarios discussed in the text. The results of each stage of Sr isotopic evolution, along with the input parameters associated with the evolutionary scenario are presented. These calculations are discussed in order in the text.

Correlation between water and ^{87}Rb/^{86}Sr in primitive meteorites

The distribution of volatile species in the early stages of evolution of the protoplanetary disk is primarily controlled by their condensation properties. Regions near the Sun are characterized by high temperatures, whereas those farther out have lower temperatures. Because solid materials are more easily retained near the Sun than gases, refractory elements are concentrated in the inner Solar System in preference to more volatile elements.

The data plotted in Figure 1 were measured by Braukmüller et al. (36) and demonstrate that the concentration of water in primitive, minimally altered meteorites, correlates with ^{87}Rb/^{86}Sr. This could, in principle, reflect either condensation properties of water, Rb, and Sr, or could be produced by secondary alteration of the meteorites in which water, Rb, and Sr are added. To

address this concern, Braukmüller et al. (36) choose minimally altered meteorites classified as grade 3. Evidence that Rb and Sr are not controlled by alteration processes is further demonstrated by Figure 1S (below) that illustrates that water content of the meteorites corelates with Rb positively and Sr negatively. This is not predicted from aqueous alteration because Rb and Sr have roughly equivalent solubilities in most fluids. Instead, the abundances of water, Rb, and Sr is consistent with condensation processes because Rb and water condensate at low temperatures, whereas Sr is a refractory element that condensates at a much higher temperature. Therefore $^{87}Rb/^{86}Sr$ of materials condensing from the protoplanetary disk serves as a reasonable proxy for more volatile species such as water.

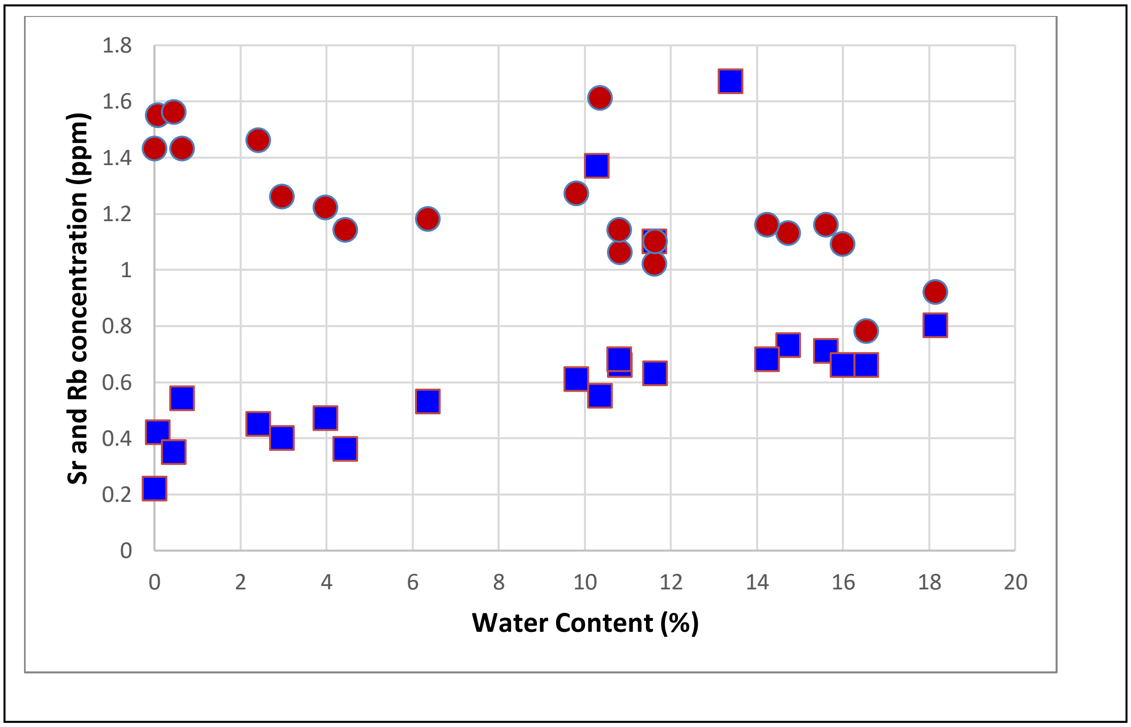

Fig. S1. Plot of Rb and Sr concentration versus H_2O contents measured in carbonaceous chondrites by (36). Rb shown in blue squares, Sr shown in red circles.

Table S1. Parameters and results of Rb-Sr isotopic evolution models discussed in the text

Model	1	2	3	4	5	6	7	8	9
Stage 1									
T_0 (Ma)	4567	4567	4567	4567	4567	4567	4567	4567	4567
I_{Sr}	0.698980	0.698980	0.698980	0.698980	0.698980	0.698980	0.698980	0.698980	0.698980
$^{87}Rb/^{86}Sr$	0.832	0.832	0.832	0.832	0.832	0.832	0.832	0.832	0.832
T_1	4565	4565	4565	4565	4565	4565	4565	4565	4565
$^{87}Sr/^{86}Sr$	0.699005	0.699005	0.699005	0.699005	0.699005	0.699005	0.699005	0.699005	0.699005
Stage 2 Proto-Earth									
T_0	4565	4565	4565	4565	4565	4565	4565	4565	4565
I_{Sr}	0.699005	0.699005	0.699005	0.699005	0.699005	0.699005	0.699005	0.699005	0.699005
$^{87}Rb/^{86}Sr$	0.081	0.019	0.832	0.0	0.130	0.081	0.031	0.037	0.025
T_1	4460	4460	4561	4520	4420	4430	4420	4420	4490
$^{87}Sr/^{86}Sr$	0.699132	0.699035	0.699564	0.699005	0.699286	0.699168	0.699072	0.699091	0.699033
Stage 2 Theia									
T_0	4565	4565	4565	4565	4565	4565	4565	4565	4565
I_{Sr}	0.699005	0.699005	0.699005	0.699005	0.699005	0.699005	0.699005	0.699005	0.699005
$^{87}Rb/^{86}Sr$	0.832	0.019	0.832	0.0	0.0	0.005	0.005	0.010	0.005
T_1	4460	4460	4561	4520	4420	4430	4420	4420	4490
$^{87}Sr/^{86}Sr$	0.700310	0.699035	0.699055	0.699005	0.699005	0.699015	0.699016	0.699027	0.699010
Stage 2 Mixing									
T:PE	1:1	1:1	1:1	9:1	9:1	9:1	7:3	9:1	9:1
$^{87}Sr/^{86}Sr$	0.699721	0.699035	0.699055	0.699005	0.699033	0.699030	0.699033	0.699033	0.699013
Stage 3									
T_0	4460	4460	4561	4520	4420	4430	4420	4420	4490
I_{Sr}	0.699721	0.699035	0.699055	0.699005	0.699033	0.699015	0.699015	0.699032	0.699013
$^{87}Rb/^{86}Sr$	0.019	0.019	0	0.019	0.019	0.019	0.019	0.019	0.019
T_1	4359	4359	4359	4359	4359	4359	4359	4359	4359
$^{87}Sr/^{86}Sr$	0.699749	0.699063	0.699055	0.699050	0.699050	0.699050	0.699050	0.699050	0.699050

Successful models reproduce the initial $^{87}Sr/^{86}Sr$ of 60025 at 4359 Ma of 0.699050. Key events for Stage 1: T_0 = start of Solar System; T_1 = Formation of Theia/Proto-Earth (dissipation of protoplanetary disk. Key events for Stage 2: T_0 = Formation of Theia/Proto-Earth; T_1 = Giant Impact. Key events for Stage 3: T_0 = Giant Impact, T_1 = crystallization age of FAS sample 60025, T:PE is the mixing ratio of Theia and proto-Earth in the Giant Impact.

Details of models are as follows:

1. Theia $^{87}Rb/^{86}Sr$ = 0.832, $^{87}Rb/^{86}Sr$ Proto-Earth = 0.081; Age Giant Impact = 4460 Ma, mixture T:PE = 1:1
2. Theia $^{87}Rb/^{86}Sr$ = 0.019, $^{87}Rb/^{86}Sr$ Proto-Earth = 0.019; Age Giant Impact = 4460 Ma, mixture T:PE = 1:1
3. Theia $^{87}Rb/^{86}Sr$ = 0.832, $^{87}Rb/^{86}Sr$ Proto-Earth = 0.832; Age Giant Impact = 4560 Ma, mixture T:PE = 1:
4. Theia $^{87}Rb/^{86}Sr$ = 0.0, $^{87}Rb/^{86}Sr$ Proto-Earth = 0.0; Age Giant Impact = 4520 Ma, mixture T:PE = 9:1
5. Theia $^{87}Rb/^{86}Sr$ = 0.0, $^{87}Rb/^{86}Sr$ Proto-Earth = 0.130; Age Giant Impact = 4420 Ma, mixture T:PE = 9:1
6. Theia $^{87}Rb/^{86}Sr$ = 0.005, $^{87}Rb/^{86}Sr$ Proto-Earth = 0.081; Age Giant Impact = 4430 Ma, mixture T:PE = 9:1
7. Theia $^{87}Rb/^{86}Sr$ = 0.005, $^{87}Rb/^{86}Sr$ Proto-Earth = 0.031; Age Giant Impact = 4420 Ma, mixture T:PE =7:3
8. Theia $^{87}Rb/^{86}Sr$ = 0.005, $^{87}Rb/^{86}Sr$ Proto-Earth = 0.037; Age Giant Impact = 4420 Ma, mixture T:PE = 9:1
9. Theia $^{87}Rb/^{86}Sr$ = 0.010, $^{87}Rb/^{86}Sr$ Proto-Earth = 0.025; Age Giant Impact = 4420 Ma, mixture T:PE = 9:1

SI References

1. G. Snyder, L. Taylor, and C. Neal, C.R., A chemical model for generating the sources of mare basalts: Combined equilibrium and fractional crystallization of the lunar magmasphere. *Geochimica et Cosmochimica Acta* **56,** 3809-3823 (1992). doi:/10.1016/0016-7037(92)90172-F.
2. J. Papike, G. Ryder, C. Shearer, Lunar samples. *Reviews in Mineralogy* **36,** 5-1–5-234 (2018). doi:/10.2138/am-1999-1001.
3. L. Borg, A. Gaffney, C. Shearer, A review of lunar chronology revealing a preponderance of 4.34–4.37 Ga ages. *Meteoritics and Planetary Science* **50,** 715–732 (2015). doi:/10.1111/maps.12373.
4. L. Nyquist, D. Bogard, C. Shih, Radiometric chronology of the Moon and Mars. In *The Century of Space Science, Chapter 55.* (eds. J. A. Bleeker, J. Geiss, and M. Huber). Kluwer, Dordrecht. pp. 1325–1376 (2001). doi:/10.1007/978-94-010-0320-9_55.
5. L. Nyquist, H, Wiesmann, B. Bansal, C. Shih, J. Keith, C. Harper, ^{146}Sm-^{142}Nd formation interval for the lunar mantle. *Geochimica et Cosmochimica Acta* **59,** 2817–2837 (1995). doi:/10.1016/0016-7037(95)00175-Y.
6. M. Boyet, R. Carlson A highly depleted moon or a non-magma ocean origin for the lunar crust? *Earth and Planetary Science Letters* **262,** 505–516 (2007). doi:/10.1016/j.epsl.2007.08.009.
7. A. Brandon, T. Lapen, V. Debaille, B. Beard, K. Rakenburg, C. Neal, Evolution and bulk Sm/Nd of the Moon. *Geochimica et Cosmochimica Acta* **73,** 6421–6445 (2009). doi:/10.1016/j.gca.2009.07.015.
8. C. McLeod, A. Brandon, R, Armytage, Constraints in the formation age and evolution of the Moon from ^{142}Nd-^{143}Nd systematics of Apollo 12 basalts. *Earth and Planetary Science Letters* **396,** 179–189 (2014). doi:/10.1016/j.epsl.2014.04.007.
9. L. Borg, A. Gaffney, T. Kruijer, N. Marks, C. Sio, J. Wimpenny, Isotopic evidence for a young magma ocean. *Earth and Planetary Science Letters* **525,** (2019). doi:/10.1016/j.epsl.2019.07.008.
10. F. Meissner, W. Schmidt-Ott, L. Ziegeler, Half-life and α-ray energy of ^{146}Sm. Zeitschrift für Physik A Atomic Nuclei (1987). **327,** 171–174 (1987). doi:/10.1007/BF01292406.
11. A. Gaffney, L. Borg, Y. Asmeron, C. Shearer, P. Burger, Disturbance of isotopic systematics during experimental shock and thermal metamorphism on a lunar basalt with implications for martian meteorite chronology. *Meteoritics and Planetary Sciences* **46,** 35-52 (2011). doi:/10.1111/j.1945-5100.2010.01137.
12. C. Alibert, M. Norman, M. McCulloch, An ancient age for a ferroan anorthosite clast from lunar breccia 67016. *Geochimica et Cosmochimica Acta* **58,** 2921–2926 (1994). doi:/10.1016/0016-7037(94)90125-2.
13. N. Marks, L. Borg, C. Shearer, W. Cassata, Geochronology of an Apollo 16 clast provides evidence for a basin-forming impact 4.3 billion years ago. *Journal of Geophysical Research: Planets* **124,** (2019). doi:/10.1029/2019JE005966.
14. L. Borg, J. Connelly, M. Boyet, R. Carlson, Evidence that the Moon is either young or did not have a global magma ocean. *Nature* **477,** 70–72 (2011). doi:/10.1038/nature10328.
15. C. Sio, L. Borg, W. Cassata, The timing of lunar solidification and mantle overturn recorded in ferroan anorthosite 62237. *Earth and Planetary Science Letters* **538,** 116219 (2020). doi:/10.1016/j.epsl.2020.116219.
16. L. Nyquist, D. Bogard, A. Yamaguchi, C. Shih, Y. Karouji, M. Ebihara, Y. Reese, D. Garrison, G. McKay, H. Takeda, Feldspathic clasts in Yamato-086032: Remnants of the lunar crust with implications for its formation and impact history. *Geochimica et Cosmochimica Acta* **70,** 5990-6015 (2006). doi:/10.1016/j.gca.2006.07.042.
17. L. Nyquist and C, -Y Shih, The isotopic record of lunar volcanism. *Geochimica et Cosmochimica Acta* **56,** 2213-2234. (1992). doi:/10.1016/0016-7037(92)90185-L.

18. R. Carlson, G. Lugmair Sm/Nd constraints on early lunar differentiation and the evolution of KREEP. *Earth and Planetary Science Letters* **45,** 123–132 (1979). doi:/10.1016/0012-821X(79)90114-6.
19. A. Gaffney, L. Borg, A young solidification age for the lunar magma ocean. *Geochimica et Cosmochimica Acta* **140,** 227–240 (2014). doi:/10.1016/j.gca.2014.05.028.
20. M. Barboni, P. Boehnke, B. Keller, I. Kohl, B. Schone, E. Young, K. McKeegan, Early formation of the Moon 4.51 billion years ago. *Sciences Advances* **3,** e1602365 (2017). doi:10.1126/sciadv.1602365.
21. L. Borg, W. Cassata, J. Wimpenny, A. Gaffney, C. Shearer, The formation and evolution of the Moon's crust inferred from the Sm-Nd isotopic systematics of highlands rocks. *Geochimica et Cosmochimica Acta* **290,** 312-320 (2020). doi:/10.1016/j.gca.2020.09.013.
22. H. Newson, Composition of the Solar System, Planets, Meteorites, and Major Terrestrial Reservoirs. A Handbook of Physical Constants *AGU Reference Shelf I* pp (1995) 159-189.
23. E. Anders, Chemical compositions of the Moon, Earth, and eucrite parent body. *Philosophical Transactions of the Royal Society of London. Series A, Mathematical and Physical Sciences*, **285,** 23-40 (1977). doi:/10.1098/rsta.1977.0040.
24. S. Taylor, Planetary Science: A Lunar Perspective. 481 pp., Lunar and Planetary Inst., Houston, (1982).
25. H. Wänke, P. Palme, H. Baddenhausen, Kruse, B. Spettel, Element correlations and the bulk composition of the Moon, *Philosophical Transactions of the Royal Society of London. Series A, Mathematical and Physical Sciences* **285**, 41-48, (1977). doi:/10.1098/rsta.1977.0041.
26. D. Anderson Chemical Composition of the Mantle. Proceedings of the fourteenth Lunar and Planetary Science Conference. *Journal of Geophysical Research* **88,** B41-B52 (1983). doi:10.1029/JB088iS01p00B41.
27. A. Ringwood Phase transformation and their bearing on the constitution and dynamics of the mantle. *Geochimica et Cosmochimica Acta* **55,** 2,083-2,110 (1991). doi:/10.1016/0016-7037(91)90090-R.
28. S. Taylor, S. McLennan, The continental crust: Its composition and evolution. Blackwell Sci. Publ., Oxford, 330 pp. (1985).
29. H. Wänke, G. Dreibus, E. Jagoutz E., Mantle chemistry and accretion history of the Earth. In Archaean geochemistry, pp. 1-24. Springer, Berlin, Heidelberg, (1984). doi:10.1007/978-3-642-70001-9_1.
30. E. Anders, N. Grevesse, Abundances of the elements: Meteoritic and solar. *Geochimica et Cosmochimica Acta* **53,** 197-214 (1989). doi:/10.1016/0016-7037(89)90286-X.
31. J. Wasson, G. Kallemeyn, Compositions of chondrites. *Philosophical Transactions of the Royal Society of London. Series A, Mathematical and Physical Sciences* **325,** 535–544 (1988). doi:/10.1098/rsta.1988.0066.
32. L. Borg, A. Gaffney, T. Kruijer, N. Marks, C. Sio, J. Wimpenny, J. Isotopic evidence for a young lunar magma ocean. *Earth and Planetary Science Letters* **523,** 115706 (2019) doi.org/10.1016/j.epsl.2019.07.008
33. L. Borg, M. Norman, L. Nyquist, D. Bogard, G. Snyder, L. Taylor, M. Lindstrom, Isotopic studies of ferroan anorthosite 62236: A young lunar crustal rock from a light rare-earth element-depleted source. *Geochimica et Cosmochimica Acta* **63,** 2679-2691(1999). doi:/10.1016/S0016-7037(99)00130-1.
34. L. Borg, J. Connelly, M. Boyet, R. Carlson, Evidence that the Moon is either young or did not have a global magma ocean. Nature 477, 70?72 (2011). doi:/10.1038/nature10328.
35. U. Hans, T. Kleine, B. Bourdon. Rb-Sr chronology of volatile depletion in differentiated protoplanets: BABI, ADOR and ALL revisited. *Earth and Planetary Science Letters* **374,** 204-214 (2013). doi:/10.1016/j.epsl.2013.05.029.
36. N. Braukmüller, F. Wombacher, D. Hezel, R. Escoube, and C. Münker, C. The chemical composition of carbonaceous chondrites: Implications for volatile element depletion, complementarity and alteration. *Geochimica et Cosmochimica Acta*. **239,** 17-48 (2018). doi:/10.1016/j.gca.2018.07.023.
